# Effects of physical and chemical factors on pseudorabies virus activity *in vitro*

**DOI:** 10.1186/s12917-020-02573-3

**Published:** 2020-09-25

**Authors:** Lang Gong, Qiwei Deng, Runda Xu, Chihai Ji, Heng Wang, Guihong Zhang

**Affiliations:** 1 College of Veterinary Medicine, South China Agricultural University, 510462 Guangzhou, China; 2 Key Laboratory of Zoonosis Prevention and Control of Guangdong Province, South China Agricultural University, 510462 Guangzhou, China; 3 National Engineering Research Center for Breeding Swine Industry, South China Agricultural University, 510462 Guangzhou, China; 4 Guangdong Laboratory for Lingnan Modern Agriculture, 510642 Guangzhou, China

**Keywords:** PRV, Biological characteristics, Physical and chemical factors

## Abstract

**Background:**

Pseudorabies (PR) is latent and can persist in infected sows for a long time, and thus, convalescent sows can carry the virus throughout life, causing severe economic losses to farmers and posing a tremendous challenge to PR prevention and control. Here, to investigate the biological characteristics of pseudorabies virus (PRV), a variety of physical and chemical factors were analyzed under controlled conditions.

**Results:**

The results showed that a high ambient temperature and dry environment led to faster virus inactivation. PRV had a certain resistance to weakly acidic or alkaline environments and was rapidly inactivated in strongly acidic or alkaline environments. The effect of ultraviolet (UV) radiation on PRV activity primarily depended on the frequency, intensity, and irradiation time of the UV exposure. Exposure to sunlight inactivated PRV via multiple factors, including temperature, sunlight intensity, UV intensity, and environmental humidity, and any shielding from sunlight strongly lowered the killing effect. Conventional disinfectants had a good disinfection effect on PRV.

**Conclusions:**

The biological characteristics of different PRV strains are variable. Generally, the activity of PRV is affected by multiple factors, which can show both synergy and antagonism. Real-world conditions should be taken into consideration to guide pork production.

## Background

Pseudorabies (PR), also known as Aujeszky’s disease or “mad itch”, is caused by pseudorabies virus (PRV), which is an enveloped DNA virus. The main susceptible animals are swine, cows, sheep, rabbits, mice, cats, and other mammals, while pigeons and chickens can resist PRV infection [1–4]. Not only are there reports of PRV isolated from people presumed to be infected with PRV but there is also direct evidence that PRV can cross hosts to infect humans [5, 6]. The amino acid positions in certain important glycoproteins, namely, gB, gC, gD and gE, may be related to the adaptability of PRV to new hosts and its immune escape from vaccines [7]. PRV was first clinically identified in cattle in 1813. After World War II, with the development of intensive farming in Europe, the number of PRV-infected pigs rose sharply. In the 1970s, PRV caused a disaster for the global pig industry due to the global flow of animals and animal products [8]. In approximately 1970, due to the large-scale outbreak of PR, many countries began to implement detection and slaughter programs. Although the United Kingdom, Denmark, Canada, the United States, and New Zealand have successfully purged PR in farm-raised swine herds, a heavy price was paid [9; 10; 11]. As people have become more aware of the disease, increasingly more countries are beginning to implement PRV eradication programs.

PRV can infect all mammals except higher primates but can only effectively replicate in swine. Therefore, swine are defined as the natural host of PRV. The nasal mucosa and oral cavity are the main sites of infection, and oral administration is more efficient than nasal inoculation [12]. PRV can also infect through vaginal mucosa or semen, which is the main mode of PRV transmission in wild boar herds. PRV can be transmitted to piglets through the placenta and colostrum. When the virus concentration in the air is high, PRV can be transmitted short distances indoors and outdoors through airflow [13]. Long-distance wind-borne transmission of PRV is still controversial. All secretions, excreta, and respiration products of infected swine, such as semen, saliva, urine, feces, and exhaled gases, contain high concentrations of PRV [14].

The resistance of infectious PRV to the environment is mainly determined by the pH, temperature, and humidity of the environment [14]. PRV has some resistance to pH in the range of 4–12. At pH values outside the critical values of 2.0 and 13.5, 2–4 h of exposure is necessary to completely inactivate PRV. In suspension, PRV may remain active for one to two months, although the duration is affected by seasonal factors. In swill undergoing lactic acid fermentation, PRV loses its activity within 24 h at 20–30 °C and can survive for 48 h and 96 h at 10℃ and 5℃, respectively. When the swill is heated to 70–80℃, the virus can only survive for 5–10 min. The virus is not inactivated in pork stored at 4℃ and can survive for approximately 40 days at -18℃ [14]. PRV can survive 15 to 40 days on hay or straw and can survive 10 and 15 days on sacks and wood products in summer and winter, respectively [14]. In addition, disinfectants, such as formalin, sodium hydroxide, trisodium phosphate iodine, quaternary ammonium salts, and chlorine preparations, are good PRV-inactivating agents [15]. Recently, germacrone was found to effectively inhibit PRV and may be used as an immunomodulator to control emerging PRV variants [16].

PR was first found over 100 years ago, and many data on its biological characteristics were published during early research on this disease. Limited by the experimental technology and equipment at that time, the data kept to date are often not sufficiently comprehensive or accurate to meet the needs of production and research. For example, selection of the optimal processing temperature and time for feed expansion or pelleting, selection of effective disinfection drugs and their application methods, determination of the quarantine period for animal introduction, and storage and transportation of laboratory biological materials are all related to the biological characteristics of PRV. In addition, the biological characteristics of the virus may have changed over the century since its first discovery, and the classic strain vaccine has not been able to provide good protection against epidemic strains. This study intends to comprehensively examine the physical and chemical factors that affect the biological characteristics of PRV and the relationships between these factors by testing the effects of various physical and chemical variables on PRV activity *in vitro*.

## Methods

### Viruses and cells

The vaccine strain Bartha K-61 (also known as PRV (K)), wild-type virulent strain PRV (B), and PK-15 cells were obtained from the College of Veterinary Medicine, South China Agricultural University. The purified wild-type virulent strain PRV (B) and the attenuated vaccine strain PRV (K) were propagated in PK-15 cells. The reproductive titer of the mutant (PRV (B)) can exceed 8 TCID_50_, while that of the classical strain (PRV (K)) can only reach approximately 6 TCID_50_.

### Effect of temperature on PRV activity

A total of 50 µL of virus solution was aliquoted into PCR tubes. A total of 23 temperature gradients were set using a PCR machine. The processing temperature range was 40–72 °C. The heat treatment time was between 0 s and 1200s. After heat treatment, each sample was inoculated into a 96-well plate with a monolayer of PK-15 cells. The cytopathic condition was observed and recorded 42 h after virus inoculation. The time at which 100% virus inactivation was observed was set as the time value. All test samples were set up in triplicate.

### Effect of pH on PRV activity

The virus was diluted 20 times in different pH solutions without buffer. The small volume of virus did not change the pH of the solution, and the pH was compared before and after treatment, with little change noted. The pH levels for exposure in this study were pH = 3, pH = 4, pH = 5, pH = 6, pH = 9, pH = 10, and pH = 11, and the treatment times were 0, 10, 20, and 30 min. The experiment was conducted in a room with a temperature in the range of 25–30 °C. The reagents used to adjust and neutralize the pH of the virus solution were 1% sodium hydroxide (NaOH), 5% NaOH, and 3.6–3.8% hydrochloric acid (HCl). After the different pH treatments, the pH value of the sample was adjusted to 7–8 with the corresponding neutralizing agent after reaching the preset treatment time.

### Effect of UV on PRV activity

PRV-containing cell culture solution was aliquoted into 33-mm-diameter plates at 200 µL per plate, the cover of the plate was opened, and the plate was irradiated with different UV intensities. The UV treatment was conducted in a biological safety hood with an ambient temperature of approximately 26 °C without ventilation. In an environment with an average C-band UV (UVC) intensity of 135 or 270 µW/cm^2^, the treatment times were 0, 1, 3, 5, 7, 9, 12, and 15 min. In an environment with non-C-band ultraviolet light, the treatment times were 0, 5, 15, 35, 60, 90, 120, 150, and 180 min.

### Effect of humidity on PRV activity

PRV cell culture solution with known titers and containing double antibiotics was plated evenly in 33-mm-diameter plates, with 50 µL per plate. Three holes with diameters of 2 mm were punched in the lid to maintain airflow with the outside. The plates were then quickly air-dried or naturally air-dried in a biological safety cabinet with a temperature of 26℃. After air-drying, three small holes with a diameter of 2 mm were punched in the lid to maintain airflow with the outside. After reaching the preset treatment time, the resulting dried residue was rehydrated. To each sample, 150 µL of Dulbecco’s modified Eagle’s medium (DMEM) containing 1% *penicillin*/*streptomycin* antibiotics was added, and the samples were then washed and mixed. The sample was inoculated into a 96-well plate along with PK-15 cells. The cells were observed for the presence or absence of cytopathic effects after 24–48 h. The control group was a sealed plate containing a known titer of PRV cell culture medium to prevent evaporation and drying of the virus solution, and the control plate was placed in a dry indoor environment.

### Effect of freeze-thawing on PRV activity

In the PRV freeze-thaw experiment, the freezing temperature was − 80 °C, and two thawing temperatures were set: 26 °C in air and 37 °C in a water bath. Diluted pseudorabies virus was aliquoted into tubes (100 µL per tube) and placed in a -80 °C freezer. The tubes were quickly taken out and thawed either in a 26 °C air environment or in a 37 °C water bath. The freeze-thaw cycles were repeated in this manner, and samples with a freeze-thaw number of 1, 5, 10, 15, 20, 25, 30, 35, and 40 were taken for titer measurement.

### Effects of storage conditions on PRV activity

PRV solution was aliquoted into 1.5 mL EP tubes at 200 µL per tube and then stored at 26℃, 4℃, -20℃, or -80℃. Samples were removed according to the experimental time points for virus titer determination, and the titer curves were drawn.

### Effects of sunlight exposure on PRV activity

A PRV stock solution of known titer was aliquoted into PCR tubes at 50 µL per tube, and the tubes were exposed to outdoor sunlight in an open and windless outside space. The control group was blocked from the sunlight with black cardboard while keeping the air flow unobstructed. Samples were taken at preset time points and were stored in the refrigerator at 4 °C for virus titer measurement. At the same time, the temperature of the experimental table, air temperature, ultraviolet intensity, and light intensity were recorded. A data logger illuminance meter (TESTES Inc, Taiwan, China) was used to measure the illumination intensity.

#### Effect of chemical disinfectants on PRV activity

The following chemical disinfectants were used in this study. Chlorine-containing disinfectant: sodium dichloroisocyanurate (SDIC), brand 84 Disinfectant. Ionic surfactants: sodium dodecyl sulfonate (SDS), powder laundry detergent, and benzalkonium bromide. Strong oxidant: potassium permanganate (KMnO_4_). Tissue fixatives: formaldehyde (HCHO) and ethanol (CH_3_CH_2_OH). Acid-base reagents: NaOH, HCl (HCl content: 36–38%), and acetic acid (CH_3_COOH).

Various chemical drugs were formulated into a 2.5% solution according to mass fraction or volume fraction, and then, a series of 2-fold dilutions were performed with distilled water. Different concentrations were obtained by dilution, and then, the dilutions were mixed with PRV solution with a known titer at a 1:1 ratio. The mixtures were allowed to stand for 30 min, which is the national standard for evaluation of the effectiveness of a disinfectant, and then inoculated at 100 µL per well into a 96-well cell plate containing a monolayer of PK-15 cells, with three replicates per gradient. The plate was then incubated at 37 °C in a 5% CO_2_ incubator for 1.5 h. The supernatant was discarded, and the plate was washed three times in phosphate-buffered saline (PBS). Next, 100 µL of fresh DMEM was added, followed by incubation for 36 h in a 37 °C incubator with 5% CO_2_. Whether cytotoxicity occurred in cells was observed and recorded.

## Results

### Effect of temperature on PRV activity

**Fig. 1:**
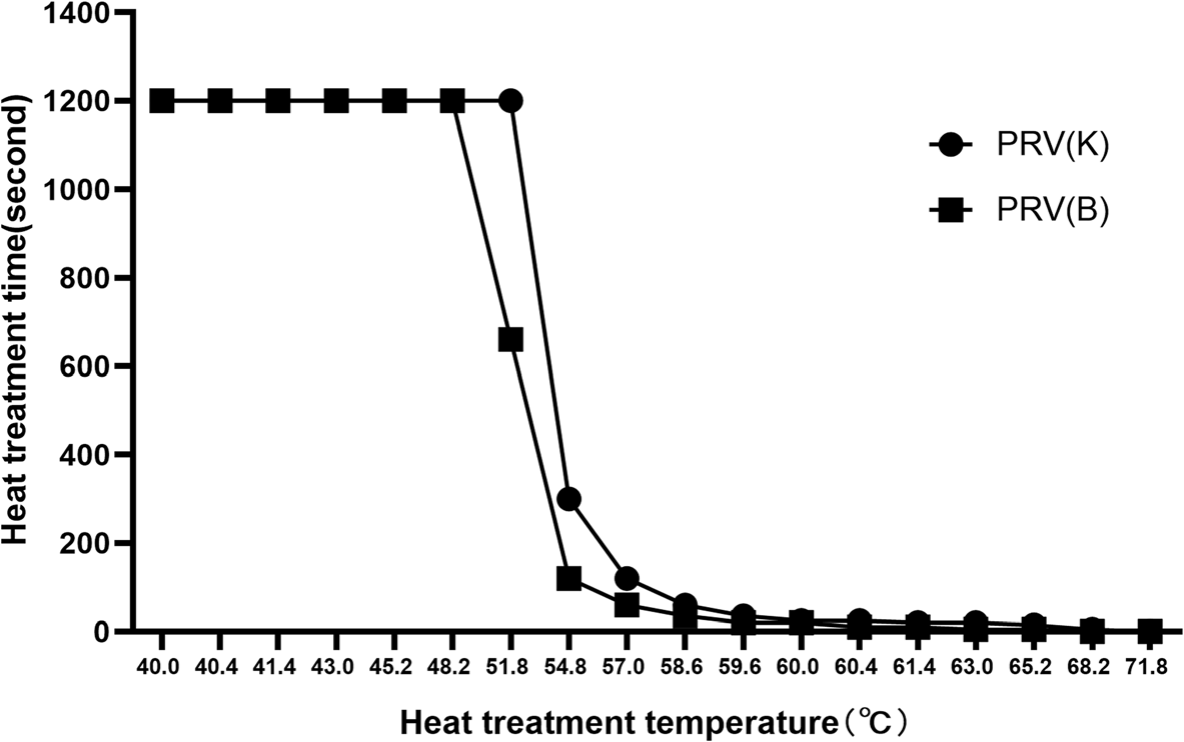
Heat resistance curve. Note: Diluted virus solution was aliquoted into 8-strip PCR tubes at 50 µL per tube. The heat treatment temperature and period were set using a PCR machine. The processing treatment temperature range was 40 °C to 80 °C, with a total of 23 temperature gradients. The heat treatment time was between 0 s and 1200s, with a total of 23 gradients. All test samples were analyzed in triplicate for each temperature and time point. After the heat treatment, 50 µL of DMEM was added to each tube. After mixing, the contents of each tube were inoculated into a 96-well plate with a monolayer of PK-15 cells. The cytopathic conditions were observed and recorded 42 h after virus inoculation. The time point was set as the time of 100% inactivation of the virus at the given temperature without any cytopathic effect (CPE)

PRV had a certain resistance to temperature, especially in a neutral liquid environment. When PRV was in the temperature range of 48–55 °C, the virus titer decreased as temperature rose, accompanied by a significant decrease in infectivity. When the temperature reached above 70 °C, PRV was inactivated within a few seconds. PRV (B) and PRV (K) had different temperature tolerances in the range of 48–60℃, with PRV (K) having a slightly stronger tolerance (Fig. 1).

### Effect of pH on PRV activity

**Fig. 2:**
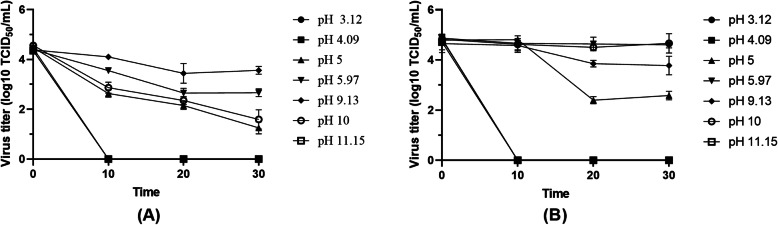
Effect of pH on PRV stability. **a**: PRV (K). B: PRV (**b**). The pH levels for exposure in this study were pH = 3, pH = 4, pH = 5, pH = 6, pH = 9, pH = 10, and pH = 11, and the treatment times were 0, 10, 20, and 30 min. The experiment was conducted in a room with a temperature in the range of 25–30 °C. Every level in the experiment was repeated three times, and the mean values are presented in the figure

A neutral liquid environment or a liquid environment with a pH of 6 to 9 had little effect on the activity of PRV, and the PRV toxicity was maintained for a long time. When PRV was in a liquid environment with a pH value of 4–6 or 9–11, the virus titer decreased slowly. When the pH value of the liquid water environment was lower than 4 or higher than 11, the PRV was rapidly denatured and inactivated, losing the ability to infect cells. The details can be seen in Fig. 2.

### Effect of UV on PRV activity

**Fig. 3:**
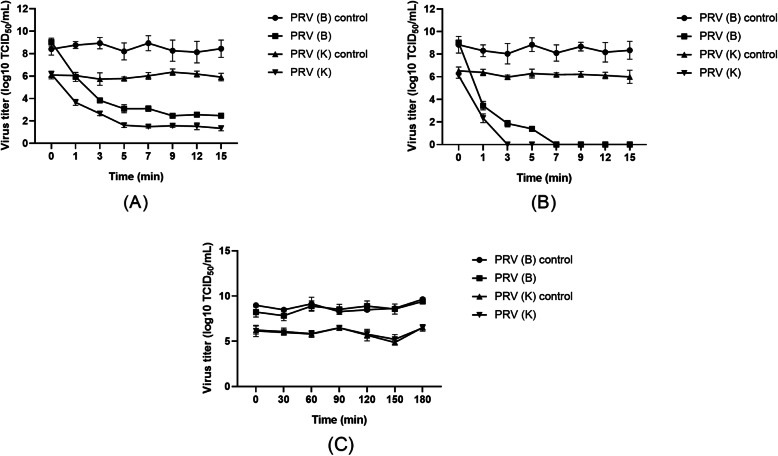
C-band UV irradiation time–titer curve. A: The average UVC intensity was 135 µW/cm^2^. B: The average UVC intensity was 270 µW/cm^2^. C: Non-C-band UV irradiation. The UV treatment was conducted in a biological safety hood with an ambient temperature of approximately 26 °C without ventilation. In an environment with an average C-band UV (UVC) intensity of 135 or 270 µW/cm^2^, the treatment times were 0, 1, 3, 5, 7, 9, 12, and 15 min. In an environment with non-C-band ultraviolet light, the treatment times were 0, 5, 15, 35, 60, 90, 120, 150, and 180 min. The viral titer in each group was determined by a TCID_50_ assay

UVC effectively inactivated PRV, while UVA or UVB did not inactivate PRV. The ability of UVC to inactivate PRV depended on the intensity and time of irradiation. The greater the intensity was, the faster the inactivation occurred, and the longer the irradiation time was, the better the inactivation effect was. PRV was completely inactivated in 7 min at an intensity of 270 µW/cm^2^. The PRV (B) strain was more resistant to UV than the PRV (K) strain (Fig. 3).

### Effect of humidity on PRV activity

**Fig. 4:**
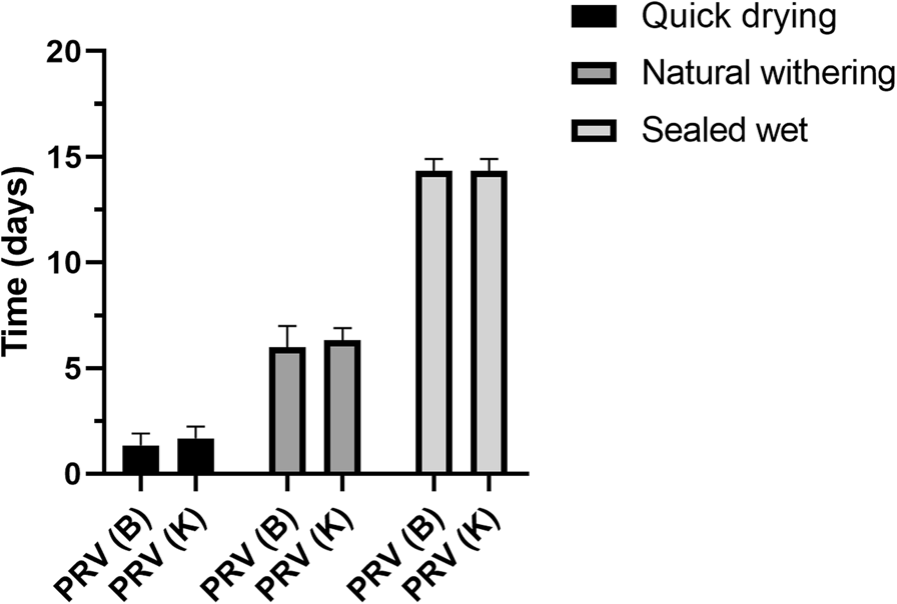
PRV survival time under different humidity conditions. Plates were quickly air-dried with a fan set to the maximum setting or allowed to air-dry naturally in a biological safety cabinet at a temperature of 26 °C. A sealed plate containing a known titer of PRV cell culture medium was set as a control. The viral titer in each group was determined by a TCID_50_ assay. The experiment was repeated three times

The activity of PRV was greatly affected by environmental humidity. A dry environment was not conducive to virus survival, and the virus activity decreased rapidly. When the virus was in a humid environment, the virus remained active for a longer time. The PRV (B) solution with a volume of 50 µL and TCID_50_ = 10^6^-10^7^/mL was dried quickly at room temperature (26℃) in a room with natural ventilation, and the infectivity was only maintained for 24 h. The infectivity of PRV (K) under the above conditions was maintained for 36 h, slightly longer than PRV (B). Both strains survived for six days after being air-dried. In a sealed and humid environment, the PRV infection ability was maintained for more than 14 days (Fig. 4).

### Effect of freeze-thawing on PRV activity

**Fig. 5:**
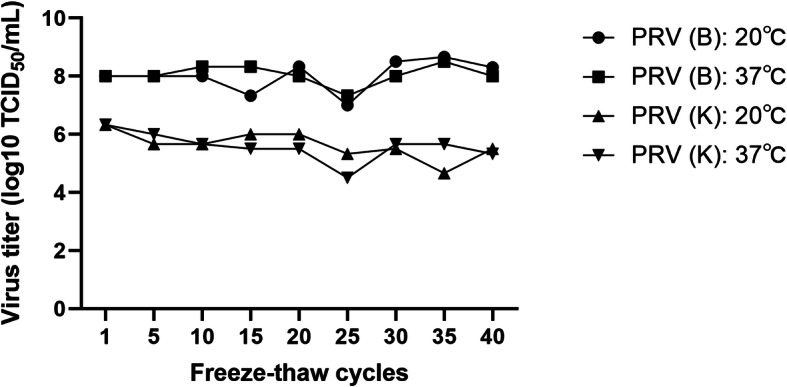
Freeze-thaw cycle–titer curve of PRV. Note: 20℃ and 37℃ represent the thawing temperatures. The freezing temperature was − 80 °C, and two thawing temperatures were set: 20 °C in air and 37 °C in a water bath. The freeze-thaw cycles were repeated in this manner, and samples with a freeze-thaw number of 1, 5, 10, 15, 20, 25, 30, 35, and 40 were taken for titer measurement

Repeated freeze-thawing had a certain effect on the activity of PRV, but the effect was not significant. Thawing temperatures of 20 °C and 37 °C did not significantly affect PRV activity. Therefore, PRV can tolerate repeated freeze-thaw cycles at certain temperatures (Fig. 5).

### Effects of different storage conditions on PRV activity

**Fig. 6:**
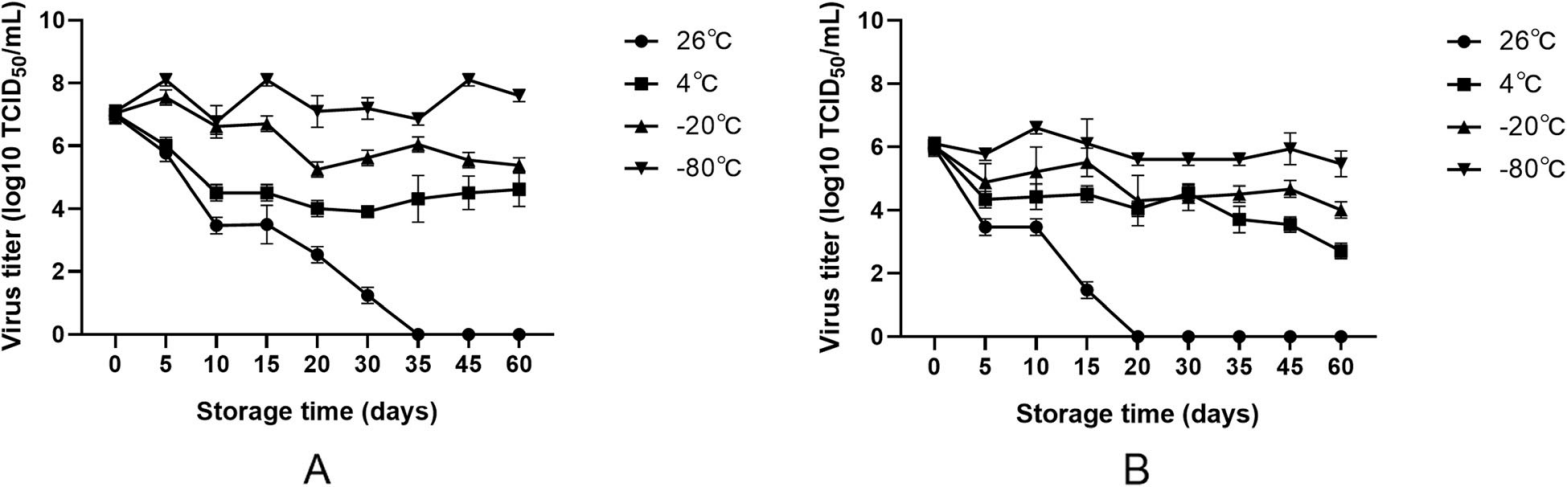
Storage time vs. virus titer curves. **a**: PRV (B). **b**: PRV (K). PRV solution was aliquoted into 1.5 mL EP tubes at 200 µL per tube and then stored at 26℃, 4℃, -20℃, or -80℃. Samples were removed according to the experimental time points for virus titer determination, and the titer curves were drawn. The experiment was repeated three times

Under short-term storage, four different storage conditions had no significant effect on the PRV titer. With long-term storage, different storage conditions had a significant effect on PRV activity. When the virus solution was stored at room temperature (26 °C), PRV (K) maintained infection ability for approximately 20 days, and PRV (B) maintained infection ability for approximately 35 days. When stored at -20 °C and − 80 °C, PRV (K) and PRV (B) viruses maintained high titers for more than 60 days. Based on analysis of the rate of titer decline, the ultralow temperature environment of -80 °C is the ideal temperature for storing PRV, which is similar to the biological characteristics of most other viruses (Fig. 6).

### Effects of sunlight exposure on PRV activity

Under direct sunlight exposure with a lowest temperature of 38 °C, a highest temperature of 39 °C, an average temperature of 38.16 °C, a lowest experimental table temperature of 38 °C, a highest experimental table temperature of 45 °C, and an average experimental table temperature of 42 °C, PRV (B) and PRV (K) lost the ability to infect cells after approximately 10 min. Under the same temperature conditions, the virus samples blocked by cardboard survived for more than 30 min. Therefore, at the same temperature, sunlight exposure will accelerate virus inactivation, and sunlight can kill PRV (Table 1).

**Table 1:** Effect of sunlight exposure on PRV activity

**Time (min)**	**Temperature (°C)**	**UVC (μW/cm**^2^**)**	**Illumination intensity(×10**^4^**Lux)**	**Viral infectivity**			
				**PRV (B)**		**PRV (K)**	
				**Direct exposure**	**Shielded**	**Direct exposure**	**Shielded**
**0**	38	31	10-11	YES	YES	YES	YES
**5**	38	30	10-11	YES	YES	YES	YES
**10**	38	30	10-11	Uncertain	YES	YES	YES
**15**	39	32	10-11	NO	YES	NO	YES
**20**	38	30	10-11	NO	YES	NO	YES
**25**	38	28	9-10	NO	YES	NO	YES
**30**	38	31	10-11	NO	YES	NO	YES

Note: The threshold for infectivity is the disposed viruses whether grow on cells or not. The cells were inoculated with virus after treatment for 48 h to investigate cell growth and the appearance of cytopathic effects

## Effect of chemical disinfectants on PRV activity

The common disinfection chemicals had good PRV disinfection ability. Among them, ionic surfactants and strong oxidants had the best disinfection effect on PRV because they had good disinfection effects at lower concentrations. Powder laundry detergent and potassium permanganate had good disinfection effects and high safety ranges (Table 2).

**Table 2:**
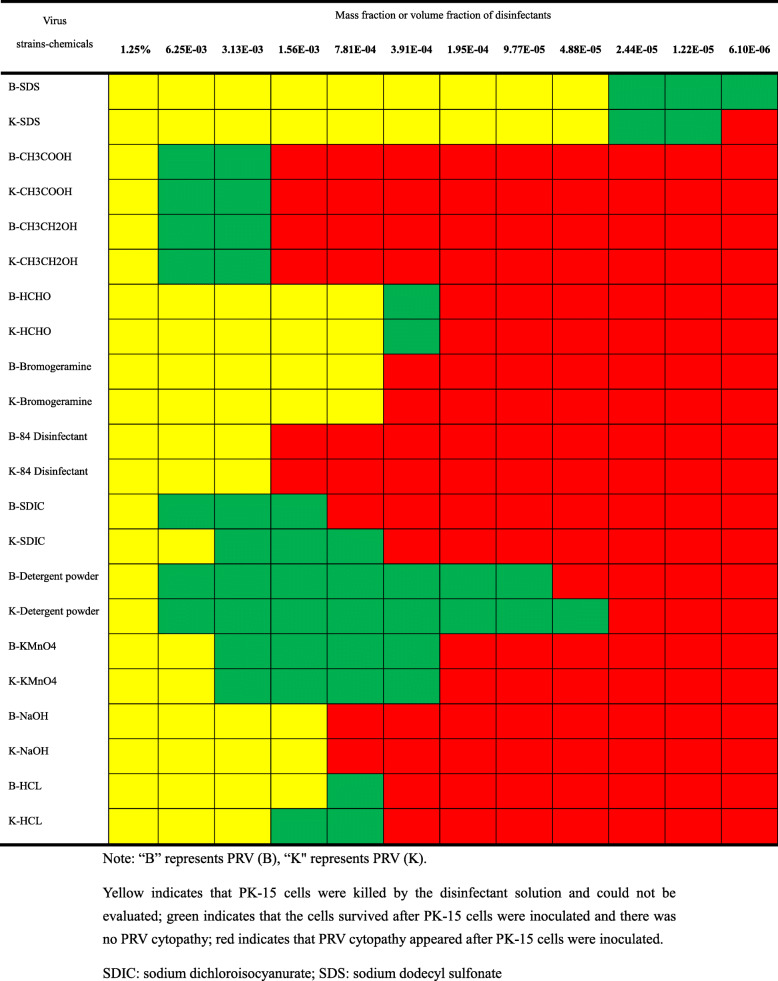
Effect of chemical disinfectants on PRV infectivity

Note: “B” represents PRV (B), “K" represents PRV (K). Yellow indicates that PK-15 cells were killed by the disinfectant solution and could not be evaluated; green indicates that the cells survived after PK-15 cells were inoculated and there was no PRV cytopathy; red indicates that PRV cytopathy appeared after PK-15 cells were inoculated. SDIC: sodium dichloroisocyanurate; SDS: sodium dodecyl sulfonate

## Discussion

At present, there are two main ways to prevent and control PR. One is to immunize swine herds with vaccines to reduce herd susceptibility; and the other is to increase the biosafety level of farms, eliminate pathogens, and cut off the spread of pathogens. Biosafety prevention and control have the advantages of low comprehensive cost and significant prevention and control effects. Such practices will be the future of prevention and control of PR and other diseases. The use of biosafety prevention and control of PRV requires an in-depth understanding of the biological characteristics of the pathogen. In addition, PRV (B) and PRV (K) are quite different in pathogenicity. We have reason to speculate that there are also differences in their biological characteristics. Therefore, this study compares the biological characteristics of different strains at the same time, laying a theoretical foundation for scientific and rational formulation of prevention and control measures.

To study the effect of temperature on the activity of PRV, many previous experiments used a water bath for heat treatment, but this method has many problems. During a pilot study of heat treatment, we found that only the temperature near the thermal sensor of the water bath was close to the chosen experimental temperature. Heat treatment of virus samples in a water bath can also easily lead to sample contamination. In the case of a large number of samples, it is difficult to accurately control the heat treatment time. When there are many samples, it places high operation standards on the experimenters, and the repeatability of the experimental data is low. To get around the above problems of heat treatment in water baths, we used a PCR machine to heat-treat virus samples. Using the PCR machine, slight differences in heat tolerance between different strains can be distinguished.

The sensitive temperature range of PRV is 50–70℃, and its activity decreases rapidly with increasing temperature. Heat resistance data have a very important guiding role in production processes. For example, when heating the feed, we can choose the optimal processing temperature and duration according to the actual production process to completely inactivate the virus and ensure achievement of multiple goals, such as no loss of nutritional content, minimum energy consumption, and reduced burden on production techniques. In addition, in terms of swine farm biosafety control, different heat treatment methods are used according to the needs of the farm to achieve thorough disinfection and save cost.

The pH value is affected by the concentration of the pH-regulating buffers and the buffering substances in the virus cell culture medium. We analyzed the rate of viral titer decline to determine the strength of the effects of different pH values on PRV activity. For example, the PRV titer decreased faster at pH = 4 than at pH = 6, and thus, the effect of pH = 4 on PRV activity is more significant than that of pH = 6. PRV is sensitive to acidic and alkaline environments. When cultivating and storing PRV, we must pay attention to control of pH. For example, when expanding PRV in the laboratory, we should choose the optimal harvest time based on the pH of the culture solution to ensure that cell metabolism does not lead to the formation of an acidic culture medium and lower the vitality of PRV. In actual production, the acid/base tolerance of PRV also plays an important role. For example, PRV is sensitive to acidic environments. Therefore, when disinfecting the environment, in addition to using conventional acid disinfectants, we can also choose acid-producing bacteria to regulate the pH of swine feed and drinking water, thereby controlling the growth and residual presence of harmful microorganisms. When studying the effect of environmental humidity on PRV activity, due to experimental error, some virus samples were contaminated with acid-producing bacteria, and thus, the virus samples became acidic. We found that virus samples contaminated with acid-producing bacteria showed a faster drop in titer than uncontaminated samples, which confirmed our hypothesis. Some highly acid-producing bacteria dropped the pH to 3.6 after fermentation at 37 °C for 24 h, the total acid production reached 35.3 g/L, and the pH dropped to below 3.6 after 48 h of fermentation. Theoretically, these acids can be used to disinfect the environment, but there are still many problems, such as which acid-producing bacteria should be selected, what the concentration of acid-producing bacteria should be, how to use them, and how to ensure efficient survival of acid-producing bacteria in the environment.

The effect of UV on PRV activity varies greatly depending on UV wavelength. UVC has the best inactivation effect on PRV but has the disadvantages of weak penetration and short killing distance. Due to the lack of instruments for measuring the intensity of UVA and UVB in the laboratory, when studying the effects of UVA and UVB on the activity of PRV, the intensity cannot be quantified, and only the power of the light source and the irradiation distance can be compared. In production, attention must be paid to UV wavelengths and intensity, the cleanliness of the air, and whether the items for disinfection are shielded or overlap with each other when UV disinfection is applied. UVC also exists in natural light. In sunny, cloudless weather, the UVC intensity is generally 20–40 µW/cm^2^. We can make full use of this natural UV light to disinfect the environment. The idea of sunlight exposure for antivirus treatment is based on this principle.

Humidity significantly affects PRV activity. For other pathogens, environmental humidity also affects pathogen spread, survival, and infection. For example, most viruses can lose the ability to infect cells when exposed to a dry environment for 36 h but can survive for more than 2 weeks in a liquid environment. Whether the viral surface antigen structure is damaged due to lack of water or whether the liquid water blocks the oxygen in the air and prevents some of the virus structures from being oxidized is not known, and the exact mechanism needs to be further investigated. During production, keeping the air humidity in the swine farm pens within a suitable range and keeping the ground, pens, and internal passages dry will help cut off the PRV transmission path and eliminate PRV from the environment.

Some disinfection agents have a single chemical structure, such as potassium permanganate, and some contain multiple chemical components, such as compound disinfectants and powder laundry detergent. Most disinfection agents have a certain effect on PRV, including ionic surfactants, strong oxidants, chlorine-containing disinfectants, and organic acids and bases. However, these disinfectants can cause harm to humans and animals and pollute the environment. When using disinfectant in actual production, it is necessary to coordinate the use of different disinfectants to achieve synergy, efficiency, economy, and environmental protection.

The influence of physical and chemical factors on PRV activity *in vitro* is often not determined by a single factor. In actual production, all factors must be fully considered in order to develop comprehensive, economical, and efficient biosafety measures to achieve scientific disinfection and prevent and control epidemic diseases.

## Conclusions

In conclusion, the activity of PRV in vitro is affected by factors such as environmental temperature, pH, UV, humidity, freeze-thawing, storage conditions and sunlight. The higher the environmental temperature, the faster the PRV inactivation, and the lower the temperature, the more conducive to the maintenance of virus activity. The greater the environmental humidity, the longer the survival time of PRV in the environment, and the drier the environment, the faster the virus inactivation. PRV has certain resistance to weak acid or weak alkaline environment, and is rapidly inactivated in strong acid or strong alkaline environment. The influence of UV on the activity of PRV is mainly related to the frequency, intensity and irradiation time of UV. The higher the frequency, the stronger the intensity, and the longer the irradiation time, the better the inactivation effect. The effect of freezing-thawing on the infectivity of PRV is not significant, and the virus can still maintain a high titer after repeated freezing-thawing. Storage environment and temperature have an impact on the infectivity of PRV. The lower the storage temperature, the slower the decrease of PRV titer. Conventional disinfectants have a good disinfection effect on PRV, and the disinfection effect is related to the concentration of the disinfectant. The higher the concentration of the disinfectant, the better the disinfection effect. Sunlight exposure can inactivate PRV, which is mainly affected by factors such as temperature, sunlight intensity, ultraviolet intensity and environmental humidity. There are differences in the biological characteristics of PRV in the natural environment. The infectivity of PRV in vitro is affected by genetic genes and environmental physical and chemical factors.

## Data Availability

The datasets used and/or analysed during the current study are available from the corresponding author on reasonable request.

## Abbreviations

PR: Pseudorabies
PRV: Pseudorabies Virus
UV: Ultraviolet
UVC: C-band UV
DMEM: Dullbecco’s Modified Eagle’s Medium
SDIC: Sodium Dichloroisocyanurate
SDS: Sodium Dodecyl Sulfonate
PBS: Phosphate-Buffered Saline
